# Superior Mesenteric Artery Syndrome—Believe in it! Report of a Case

**DOI:** 10.1155/2012/282646

**Published:** 2012-08-05

**Authors:** Sante Capitano, Gianfranco Donatelli, Gianfranco Boccoli

**Affiliations:** ^1^Dipartimento di Chirurgia e Patologia Chirurgica, Istituto di Ricovero e Cura a Carattere Scientifico (INRCA), P.O.R. Ancona, Via Della Montagnola 81, 60100 Ancona, Italy; ^2^Nouvel Hopital Civil, Les Hopitaux Universitaires de Strasbourg, IRCAD-EITS, 67000 Strabourg, France

## Abstract

We present a case of a man with amyotrophic lateral sclerosis who developed superior mesenteric artery syndrome (SMAS) following the confection of feeding jejunostomy. He was successfully managed by conservative treatment. Left lateral positioning during enteral feeding allowed quick resolution of the occlusive state. Various surgical interventions have been associated with SMAS, directly or indirectly, by reducing the width of the aortomesenteric angle. The operative stress was probably what triggered symptomatology in our patient thus to conclude that the surgical stress should be considered as a causal factor triggering the SMAS in a context of other predisposing factors.

## 1. Introduction

Superior mesenteric artery syndrome (SMAS) is a rare cause of small bowel obstruction, characterized by an extrinsic vascular compression of the third portion of the duodenum between the abdominal aorta and overlying superior mesenteric artery, due to loss of cushion of fat. Clinically, it presents with postprandial abdominal distension, pain, nausea, vomiting, and weight loss. Fasting, total parenteral nutrition, and gastric decompression constitute usual conservative treatment with a high success rate. Surgery if needed, has a low failure rate and consists of creating a gastrojejunostomy or duodenojejunostomy with or without duodenal mobilization (known as the Strong's procedure).

Here, we present the case of a man suffering from amyotrophic lateral sclerosis (ALS) who developed SMAS following the confection of feeding jejunostomy done for significant malnutrition.

## 2. Case Presentation

A 57-year-old man, suffering from ALS since 2009 with concomitant marked lumbar lordosis and quadriplegia, was admitted for marked disease progression, respiratory distress, and complete dysphagia (for both liquids and solids).

Tracheostomy was initially performed and the patient was placed on mechanical ventilation. Given the significant malnutrition, percutaneous endoscopic gastrostomy was attempted but failed due to technical reasons. Then a surgical jejunostomy with Witzel technique was performed.

The post operative course was complicated by vomiting and gastric distension treated with nasogastric tube placement that drained approximately 700–1500 mL of biliary secretions per day.

**Figure 1:**
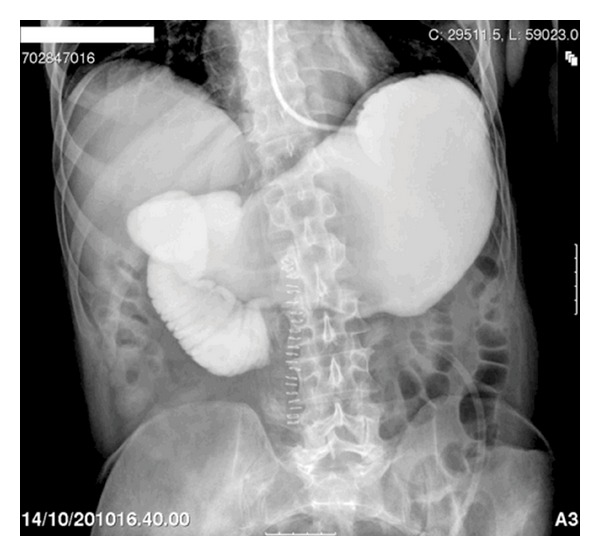
Upper oral contrast series showing contrast stop point in duodenum.

Upper oral contrast series were then performed showing some contrast stagnation at the level of the third part of duodenum (Figure 1). The patient did not have any pain or abdominal signs. Biochemical markers were in the normal range. The jejunostomy was functional.

**Figure 2:**
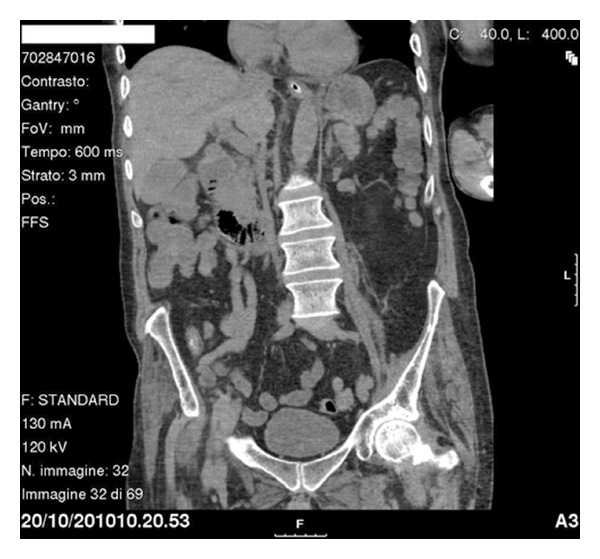
Coronal CT image showing duodenal clamping.

**Figure 3:**
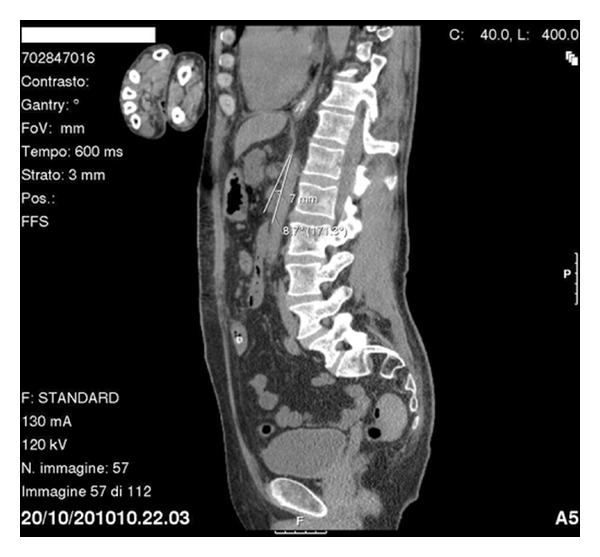
Sagittal CT image demonstrating the aortomesenteric angle.

 A prokinetic medical treatment with prostigmin and erythromycin was then started resulting in intermittent occlusion. We then decided to perform a CT scan in order to achieve better visualisation of the duodenal stop. It showed third part of duodenum completely clamped between the aorta and superior mesenteric artery, with a marked narrowing of the aortomesenteric angle to 8.7 degrees measuring 7 mm (Figures 2 and 3). These findings pointed towards diagnosis of SMAS and a conservative treatment was established with total enteral nutrition to 80 mL/h done in left lateral position. The nasogastric tube was withdrawn three days later and the patient was discharged from the hospital one week after with no occlusive symptoms. At followup of three weeks, he reported weight gain and could stand in supine position without presenting any abdominal symptoms.

## 3. Discussion

Superior mesenteric artery syndrome was described for the first time by Rokitansky in 1842 and has an incidence of 0.013–0.3% in the general population with a mortality rate of 33% [1, 2]. Other names also used to indicate this clinical entity include Wilkie's syndrome [3], aortomesenteric artery compression [4], arteriomesenteric duodenal compression [5], duodenal vascular compression [6], and cast syndrome [6].

It occurs more frequently in women and clinically presents with vomiting, nausea, postprandial abdominal distension, pain, and weight loss [7]. Other patients suffer from nonspecific symptoms for months to years [2, 3]. It is caused by mechanical compression of one of the most fixed parts of the duodenum (third), between the aorta and the origin of superior mesenteric artery. Normally this angle is between 36–56 degrees with an opening of 10–20 mm, which, in the case of this syndrome, get reduced to between 6–15 degrees for the angle and 2–8 mm for aortomesenteric aperture [8, 9].

Aortomesenteric angle's width is related to the body mass index [10].

Lack of retroperitoneal and periduodenal fat pads can lead to a more acute angle resulting in duodenal “clamping.”

SMAS is thus triggered by any condition compromising the normal fat cushions and the mesenteric angle. Fat cushion loss can be seen in catabolic and postoperative states, in the presence of congenital anomalies or trauma.

Various surgical interventions have been associated with SMAS, directly or indirectly, by reducing the width of the aortomesenteric angle. Examples include bariatric surgery, scoliosis surgery, ileoanal pouch anastomosis, and aortic aneurysm repair [11].

In our case, significant weight loss due to disease progression and lumbar lordosis was not accompanied with clinical symptoms of high digestive obstruction in the preoperative period.

Postoperatively however the coexistence of a second pathology was suspected, but CT scan subsequently excluded other possible pathologic diagnosis.

He was successfully managed by conservative treatment. Left lateral positioning during enteral feeding allowed quick resolution of the occlusive state.

This phenomenon can be explained that the mesentery and small bowel move to the left from right side of the abdomen opening the aortomesenteric angle. In the presence of the jejunostomy totally enteral hypercaloric nutrition was started.

Some authors report that weight gain does not contribute to retroperitoneal fat accumulation and that deposition of fat does not play a role in the resolution of SMAS [12].

In our case the adequate positioning only temporarily solved the problem, three week later, only after gaining weight, the patient was completely relieved from the symptoms.

The operative stress was probably what triggered symptomatology in our patient, since clinical signs of high-level occlusion only appeared afterwards.

Surgery is indicated for symptomatic patients when conservative treatment fails. There are no guidelines concerning the precise duration of the conservative treatment; a 169-day conservative treatment is the longest one reported [13–15]. We feel that SMAS may be far more prevalent and is under-diagnosed. So it should be taken into consideration in all patients with important weight loss and gastric distension. Secondly, surgical stress should be considered as a causal factor triggering the SMAS in a context of other predisposing factors. In conclusion we would like to point out the difficulty in achieving the accurate diagnosis of SMAS in a clinical setting similar to the one presented here, and the importance of excluding different surgical pathologies in the early setting.
